# Altered dynamic functional and effective connectivity in drug-naive children with Tourette syndrome

**DOI:** 10.1038/s41398-024-02779-1

**Published:** 2024-01-22

**Authors:** Lekai Luo, Yi Liao, Fenglin Jia, Gang Ning, Jing Liu, Xuesheng Li, Xijian Chen, Xinmao Ma, Xuejia He, Chuan Fu, Xiaotang Cai, Haibo Qu

**Affiliations:** 1 Department of Radiology, West China Second University Hospital, Sichuan University, Chengdu, 610021 Sichuan PR China; 2 Key Laboratory of Birth Defects and Related Diseases of Women and Children (Sichuan University), Ministry of Education, Chengdu, 610021 Sichuan PR China; 3 Department of Rehabilitation, West China Second University Hospital, Chengdu, 610021 Sichuan PR China

**Keywords:** Neuroscience, Psychiatric disorders

## Abstract

Tourette syndrome (TS) is a developmental neuropsychiatric disorder characterized by repetitive, stereotyped, involuntary tics, the neurological basis of which remains unclear. Although traditional resting-state MRI (rfMRI) studies have identified abnormal static functional connectivity (FC) in patients with TS, dynamic FC (dFC) remains relatively unexplored. The rfMRI data of 54 children with TS and 46 typically developing children (TDC) were analyzed using group independent component analysis to obtain independent components (ICs), and a sliding-window approach to generate dFC matrices. All dFC matrices were clustered into two reoccurring states, the state transition metrics were obtained. We conducted Granger causality and nodal topological analyses to further investigate the brain regions that may play the most important roles in driving whole-brain switching between different states. We found that children with TS spent more time in state 2 (*P*_FDR_ < 0.001), a state characterized by strong connectivity between ICs, and switched more quickly between states (*P*_FDR_ = 0.025) than TDC. The default mode network (DMN) may play an important role in abnormal state transitions because the FC that changed the most between the two states was between the DMN and other networks. Additionally, the DMN had increased degree centrality, efficiency and altered causal influence on other networks. Certain alterations related to executive function (*r* = –0.309, *P* < 0.05) and tic symptom ratings (*r* = 0.282; 0.413, *P* < 0.05) may represent important aspects of the pathophysiology of TS. These findings facilitate our understanding of the neural basis for the clinical presentation of TS.

## Introduction

Tourette syndrome (TS) is a developmental neuropsychiatric disorder characterized by repetitive, stereotyped, involuntary motor and vocal tics lasting for at least 12 months, affecting 1–3% of children [1, 2]. Tic symptoms generally appear around the ages of 6–7 years, and symptom severity can increase with age, often peaking in intensity in early adolescence [3]. TS is strongly comorbid with other developmental neuropsychiatric disorders, especially attention deficit hyperactivity disorder (ADHD) (50% of children with TS also have ADHD) and obsessive-compulsive disorder (OCD) (~20–60% of children with TS also have OCD) [4]. However, the underlying pathophysiology of TS remains controversial and poorly understood. TS has been hypothesized to be associated with cognitive difficulties involving executive dysfunction in the frontal cortex (e.g., prefrontal cortex, anterior cingulate cortex, dorsolateral frontal areas) including response inhibition and selective attention [5, 6]. Brain dopamine and 5-hydroxytryptamine dysregulation and their interactions are currently considered to be related to tics, and abnormalities in cerebral cortex-striatum-thalamus-cortex (CSTC) circuits are associated with the development of TS [7].

Altered structural and functional connectivity (FC) is considered a key contributor to psychopathology [8–10]. There is a growing tendency to understand developmental neuropsychiatric disorders including TS as a consequence of “brain network dysfunction”. Over the past few decades, advanced neuroimaging methods have been used to investigate the neurological basis of TS. A task-based functional MRI (fMRI) study found that the supplementary motor area appears to be overactive in children with TS when performing motor tasks [11]. Both fMRI and magnetoencephalography studies have shown increased FC between the supplementary motor area and motor cortex in chronic tic patients [12, 13]. A meta-analysis of task-based neuroimaging studies on TS showed that children with TS have abnormal activation of the motor, sensory, prefrontal, anterior cingulate, and temporoparietal association cortices [14]. Previous static resting state fMRI (rfMRI) studies have shown widespread abnormalities in the sensorimotor network (SMN) and fronto-parietal network (FPN) of children with TS [15, 16]. Some studies have examined common and specific alterations of static FC among TS and other comorbidities. Specially, Openneer et al. found local efficiency and clustering coefficient were significantly lower in children with pure TS in the default mode network (DMN) when compared to healthy controls, and in the frontoparietal network compared with children with pure ADHD [16]. Tikoo et al.’s [17] study showed that patients with pure TS and patients with composite of TS and OCD exhibited similar FC alteration in contrast with controls, while pure patients with OCD showed distinctive patterns of FC changes prominently involving the cerebellum and the frontoparietal network. These studies have demonstrated that patients with TS who have comorbidities or patients with pure ADHD or OCD have shared and specific FC patterns compared with pure TS [16–18].

Conventional rfMRI FC studies treat brain connectivity patterns as stationary during scans. However, the brain is a highly dynamic system with rapidly changing neural interactions and nonstationary neural activities [19], and this valuable information is lost in static FC analysis. dFC fluctuations are related to changes in affective and executive processes [20], and altered dFC has been proved to be associated with changes in brain functions whose alterations characterize psychiatric disorders [21]. In some cases, dFC features provide higher predictive accuracy for diagnostic and prognostic purposes than static FC metrics [22]. The potential biomarker utility and clinical relevance of dFC patterns have been demonstrated in several psychiatric disorders. Recently, Xin et al. [23] explored abnormal brain dynamic FC in 36 boys with TS compared with 27 healthy controls, which is the first dynamic FC study about TS. They found children with TS have higher fractional time and dwell time in the state characterized by increased inter-network connections, and the state transition metrics and dynamic topological metrics associated with tic symptom severity. Their study highlights the novel insights that altered brain functional dynamics may contribute to the neuropathology of TS.

Several approaches have been developed to calculate dynamic properties of the brain. The sliding-window method is the most widely used [24]. Each windowed FC matrix can be assigned into a specific pattern named “state,” which represents a specific brain network configuration [25]. After obtaining the FC states, we wonder in the resting state, which brain regions drive the whole brain switches among different states. Effective connectivity (EC) measures the effect of one brain region on another brain region in a particular direction, which provides information closely related to the causal processes that how one brain region influence another. Granger causality analysis (GCA) is a widely used computational method to calculate the EC of neural networks [26]. We suspect that abnormal EC may be the underlying cause of abnormal state transitions in children with TS. We then calculated the degree centrality and other topological metrics of each brain region. Brain regions with high degree centrality have high “importance” in the brain because of their wide connection with the rest of the brain.

In the present study, we used rfMRI and the sliding-window approach to identify abnormalities in the dynamic FC characteristics of children with TS. We then calculated the EC and nodal graph theory metrics to identify the brain regions that may drive whole-brain switches among different states over time. Finally, we explored whether altered dynamic FC, EC, and nodal topological characteristics are significantly associated with the clinical features of children with TS. We hypothesized that children with TS would show abnormal temporal properties of dFC states, brain regions with altered EC and degree centrality may play important roles in state transitions, and these abnormal brain functional measures would be associated with executive function and symptom severity in children with TS.

## Materials and methods

### Participants

Participants were recruited from July 2015 to June 2020 at West China Second Hospital of Sichuan University. This study was approved by the local research ethics committee of West China Second Hospital of Sichuan University, and informed written consent was obtained from the legal guardians of all participants. A total of 75 drug-naive children with TS and 68 typically developing children (TDC) were recruited for our study. TS diagnosis was determined by experienced pediatric neurologist using the Diagnostic and Statistical Manual of Mental Disorders-5 (DSM-V) with the following criteria: (1) both multiple motor and one or more vocal tics were present at some time during the illness, although not necessarily concurrently; (2) the tics may wax and wane in frequency, but have persisted for more than one year after the onset of the first tic; (3) the disturbance is not due to the direct physiological effects of a general medical condition (e.g., Huntington’s disease or post-viral encephalitis) or a substance (e.g., cocaine); and (4) the onset of illness occurred before 18 years of age. TDC were screened using the DSM-V Non-Patient Edition to exclude individuals with Axis I psychiatric diagnoses. Exclusion criteria for all participants included: (1) other psychiatric diseases; (2) significant systemic or neurological illness or neurosurgery; (3) claustrophobia or any other MR contraindications; and (4) maximum head displacement >3 mm or maximum rotation >3° during rfMRI scanning. The MR images were inspected by experienced neuroradiologists to confirm the absence of gross brain abnormalities. The clinical data of the participants were assessed using a standardized process for assessment and collection by clinicians. The Yale Global Tic Severity Scale was used to assess tic severity [27]. The Wisconsin Card Sorting Test (WCST) measured the executive function involving inhibitory control and cognitive flexibility [28].

After data acquisition and quality control, the final sample comprised 54 children with TS and 46 TDC. There was no differences in age, sex and head motion between the two groups (Table 1). Moreover, there was no significant correlation between head motion and clinical characteristics in children with TS (Table S1).

**Table 1:** Demographic and clinical characteristics of study participants.

	TS (*n* = 54)	HC (*n* = 46)	χ^2^/*t* value	*P* value
Sex (number)	40 M, 14 F	29 M, 17 F	1.413	0.235
Age (years)	8.3 ± 2.1 (4–14)	8.9 ± 2.8 (4–14)	−1.162	0.248
Duration (months)	20.2 ± 25.7 (0–120)	-	-	-
Head motion (mean FD)	0.162 ± 0.114	0.137 ± 0.094	1.219	0.226
Total YGTSS score	44.0 ± 16.0	-	-	-
Total motor tic score	13.0 ± 5.3	-	-	-
Number of motor tic	2.3 ± 0.9	-	-	-
Frequency of motor tic	3.3 ± 0.8	-	-	-
Strength of motor tic	3.1 ± 0.7	-	-	-
Complexity of motor tic	2.8 ± 1.0	-	-	-
Interference of motor tic	2.8 ± 0.9	-	-	-
Total phonic tic score	5.4 ± 5.2	-	-	-
Number of phonic tic	1.1 ± 0.3	-	-	-
Frequency of phonic tic	2.5 ± 0.9	-	-	-
Strength of phonic tic	2.5 ± 0.7	-	-	-
Complexity of phonic tic	1.5 ± 0.6	-	-	-
Interference of phonic tic	2.0 ± 0.8	-	-	-
WCST total number of correct	35.1 ± 9.6	-	-	-

Values were given as mean ± SD (range). *P* value of sex was obtained by chi-square test and *P* values of age was obtained by two-sample *t* test.

*M* male, *F* female, *TS* Tourette’s syndrome, *HC* healthy control, *YGTSS* Yale Global Tic Severity Scale, *WCST* Wisconsin Card Sorting Test.

### MR data acquisition and preprocessing

All participants underwent rfMRI using a 1.5-T Philips MRI system. rfMRI data were obtained using a gradient-echo echo-planar imaging sequence with the following parameters: repetition time (TR), 3000 ms; echo time (TE), 50 ms; flip angle, 90°; slice thickness, 4 mm; field of view, 230 × 230 mm^2^; matrix size, 64 × 64; and voxel size, 3.59 × 3.59 × 4.00 mm^3^. Each brain volume was comprised of 30 axial slices to cover the whole brain, and each functional imaging session contained 150 volumes. During scanning, the participants were instructed to keep their heads still and relax with their eyes closed, without falling asleep or having systematic thoughts. Earplugs and foam padding were used to reduce the noise and head motion.

Preprocessing was performed using a toolbox for Data Processing & Analysis of Brain Imaging (rfmri.org/DPABI) with a standardized protocol (Supplementary methods). To eliminate the influence of head motion, participants with a maximum displacement of >3 mm and maximum rotation of >3° were excluded from further analysis.

### GICA and identification of independent components (ICs)

Group independent component analysis (GICA) is a data-driven approach for decomposing rfMRI data into functionally homogeneous regions [29]. We performed spatial GICA using the GIFT toolbox (Mialab.mrn.org/software/gift). The number of ICs was estimated to be 21, and nine ICs were identified as meaningful based on specific criteria [30] (see details in the Supplementary methods).

**Fig. 1:**
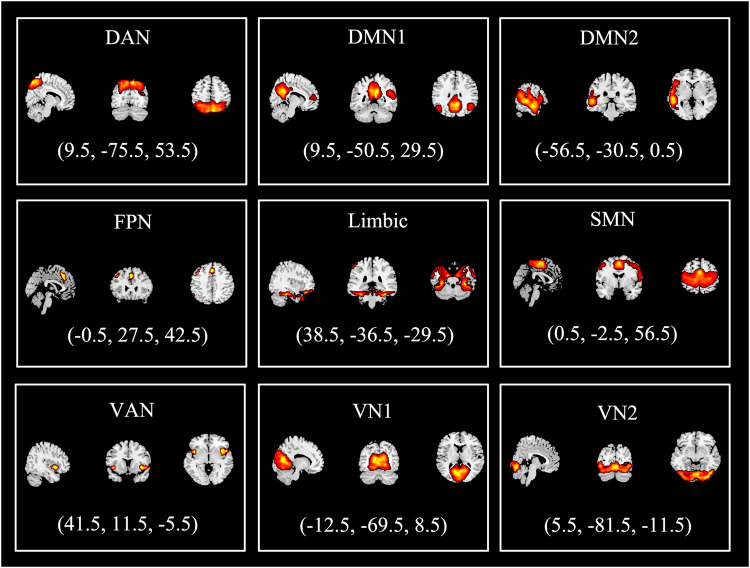
Nine selected independent components (ICs) for all participants in this study. Each IC represents an intrinsic brain functional network. These networks included the dorsal attention network (DAN), default mode network (DMN), frontoparietal network (FPN), limbic network, sensorimotor network (SMN), ventral attention network (VAN), and visual network (VN).

Based on Yeo’s seven functional brain network templates [31], we sorted the nine selected ICs into different functional networks (Fig. 1): dorsal attention network (DAN), DMN, FPN, limbic network, SMN, ventral attention network (VAN), and visual network (VN). The sorting rules is shown in the Supplementary methods. We also used the Stanford functional ROI template [32] and the Anatomical Automatic Labeling templates to replicate and validate the analysis.

### dFC state analysis

**Fig. 2:**
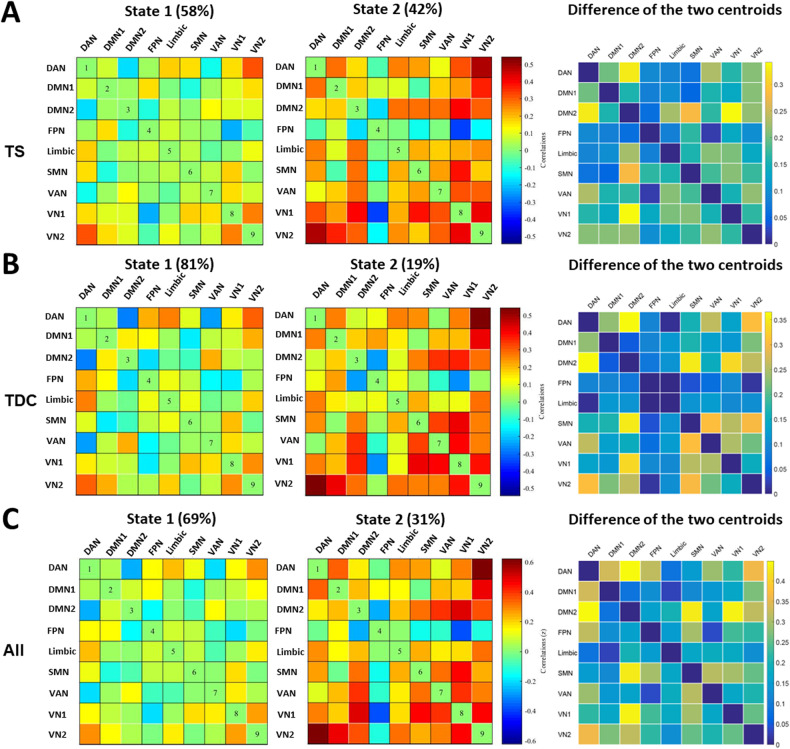
Group specific cluster centroids and cluster centroids for all participants under 20-TR window size. **A** Cluster centroids of state 1 (the left) and state 2 (the middle) for Tourette’s syndrome (TS) group, as well as the absolute value of the difference between the two centroids (the right). **B** Cluster centroids of state 1 (the left) and state 2 (the middle) for typically developing children (TDC) group, as well as the absolute value of the difference between the two centroids (the right). **C** Cluster centroids of state 1 (the left) and state 2 (the middle) for all participants, as well as the absolute value of the difference between the two centroids (the right).

dFC was examined using the temporal dFNC toolbox in the GIFT (see details in the Supplementary methods). A sliding-window approach was used to explore time-varying changes in FC within the nine ICs identified during the rfMRI scans. We chose a 20-TR window (60 s) because previous studies have suggested that windows of 30–60 s can successfully capture the patterns of resting-state fluctuations in the dFC [24]. To assess recurring dFC patterns, k-means clustering was performed on the FC matrices of all the time windows for all individuals. The optimal k value was determined to be two (Fig. S1). We used the cluster centroids of all the participants to represent the two recurring FC states (Fig. 2C). To visualize the dFC states in the two groups, we estimated group-specific cluster centroids (Fig. 2A, B). Group specific cluster centroids and cluster centroids for all participants under 15-TR window size are similar with that under 20-TR window size (Fig. S2). To examine the temporal properties of the dFC states, we assessed three different state characteristics: (1) the fractional time, which indicates the proportion of the time window belonging to each state; (2) the mean dwell time, which represents the average length of time spent in each state (measured number of consecutive time windows) before switching to other states; and (3) the number of transitions, which represents the number of switches among states over time. To ensure consistency and validity of the dFC state analysis at different window sizes, we used 15-TR to 19-TR window size to repeat the above analysis and validate the results.

### Effective connectivity: multivariate GCA

Multivariate GCA (mGCA) was performed to assess EC among the 9 ICs using a vector autoregression model in the REST-GCA toolbox (http://www.restfmri.net/forum/) based on Matlab. This technique obtains a directed transfer function from a multivariate autoregressive model of the time series of the selected region of interests (ROIs) and further detects the causal relationship between any two ROIs, which may be mediated by a third ROI. The signed strength and direction of the relationships between each pair of ICs were obtained. Positive signed-path coefficients indicate that the past activity of a brain region can predict the increased activity of another brain region. In other words, positive coefficients from X to Y indicate that the activity in region X exerts a causal influence on the activity in region Y in the same direction (i.e., a positive influence). Similarly, the negative coefficients from X to Y suggest that the activity of brain region X exerts an opposing directional influence on the activity of region Y (i.e., a negative influence). In the current study, Granger causality strength characterized signed-path coefficients and the direction of the relationship between each pair of regions, indicating a directional (X to Y or Y to X) excitatory or inhibitory effect among the brain networks. Finally, the 9 × 9 asymmetric adjacency matrix (Granger causality pattern) was calculated for each participant.

### Nodal topological metrics for each IC

To measure the “importance” of each IC function in the whole brain, we applied a graph theory approach to examine the degree centrality of each IC. We preserved only positive FCs and used weighted connectivity strength to calculate the degree centrality. The degree centrality of each IC was measured as the sum of the weighted connectivity strengths between the selected IC and all the other ICs of the brain. Other nodal graph theory metrics including nodal betweenness centrality, clustering coefficient, efficiency, and local efficiency were also calculated. The mathematical definitions and interpretations of these topological network measures are shown in Table S2.

### Statistical analysis

The nonparametric permutation approach (10,000 iterations) was used to test for group differences in all the state transition metrics owing to their non-normal distribution. A two-sample *t* *test* was used to measure the between-group differences in effectiveness and tolological metrics. Significance was set at *P* < 0.05, with false discovery rate (FDR) correction for multiple comparisons. We used the “mafdr” function in the matlab to do the FDR correction, and applied FDR correction for each analytical step.

To explore the relationship between altered brain function and clinical symptom severity in the TS group, we performed partial correlation analyses using age and sex as covariates. Nominal significance was set at *P* < 0.05 for these correlation analyses conducted for heuristic and descriptive purposes. To exclude the potential influence of head motion, we used head motion as an additional covariate to perform the correlation analyses.

## Results

### dFC state analysis

Two recurring brain functional states were defined by clustering windows based on the spatial similarity of the dFC matrices, and their centroids are shown in Fig. 2. State 1 was characterized by weak connectivity between ICs, whereas state 2 had stronger and mostly positive connectivity. The proportion of state 1 (69.0%) was higher than that of state 2 (31.0%) across all participants. For children with TS, the total occurrence of state 1 was 58% and the total occurrence of state 2 was 42%. For TDC, the total occurrence of states 1 and 2 was 81% and 19%, respectively.

**Fig. 3:**
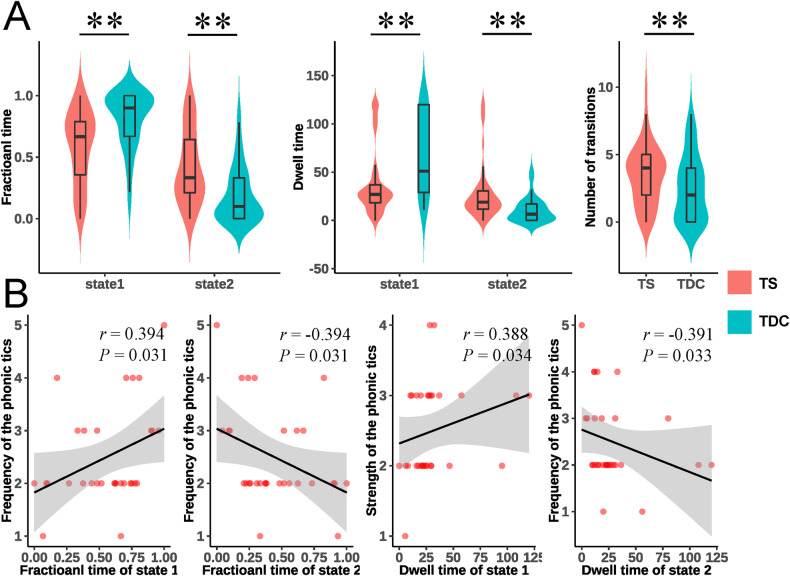
Statistical analysis for state transition metrics. Group comparisons of state transition metrics (**A**), and correlations between state transition metrics and tic symptom ratings (**B**) in children with TS. The “**” represents *P*_FDR_ < 0.05.

Group comparisons of state transition metrics (Fig. 3A) showed that children with TS had decreased fractional time in state 1 (*P* < 0.001, *P*_FDR_ < 0.001) and increased fractional time in state 2 (*P* < 0.001, *P*_FDR_ < 0.001) compared to TDC. We also found that children with TS exhibited shorter dwell time in state 1 (*P* < 0.001, *P*_FDR_ < 0.001) and longer dwell time in state 2 (*P* < 0.001, *P*_FDR_ < 0.001) than those with TDC. Moreover, we observed a higher number of transitions in children with TS than in those with TDC (*P* = 0.025, *P*_FDR_ = 0.025). The results of the validation analysis using different window size (Fig. S3 and Tables S3–1 to S3-5) and different templates (Figs. S4 and S5) were similar to the main results (see details in the Supplementary results).

In the correlation analysis, we found that the frequency of phonic tics was positively correlated with the fractional time of state 1 (*r* = 0.394, *P* = 0.031) and negatively correlated with the fractional time of state 2 (*r* = – 0.394, *P* = 0.031) in the TS group. We also found that the dwell time of state 1 was positively correlated with the strength of phonic tics (*r* = 0.388, *P* = 0.034), while the dwell time in state 2 was negatively correlated with the frequency of phonic tics (*r* = – 0.391, *P* = 0.033) (Fig. 3B). Other clinical scores and duration didn’t show significant correlation with state transition metrics. After accounting for head motion as a covariate, we found similar correlation results to the primary results (Table S4).

To characterize the most prominent alteration from state 1 to state 2, we calculated the extent of change between the two states (measured by absolute value of the FC difference between the two cluster centroids). Since this step aims to focus on the extent of changes in communication between brain regions, we chose to use the absolute value of the difference between the two states irrespective of whether the direction of the change was increased or decreased. We supposed these FC changed most between the two states would be essential for state transitions. We found that the FC that changed the most from state 1 to state 2 included the FC of the DMN-DAN, SMN-DMN, and VN-DMN (Fig. 2, the right column).

### Effective connectivity

**Fig. 4:**
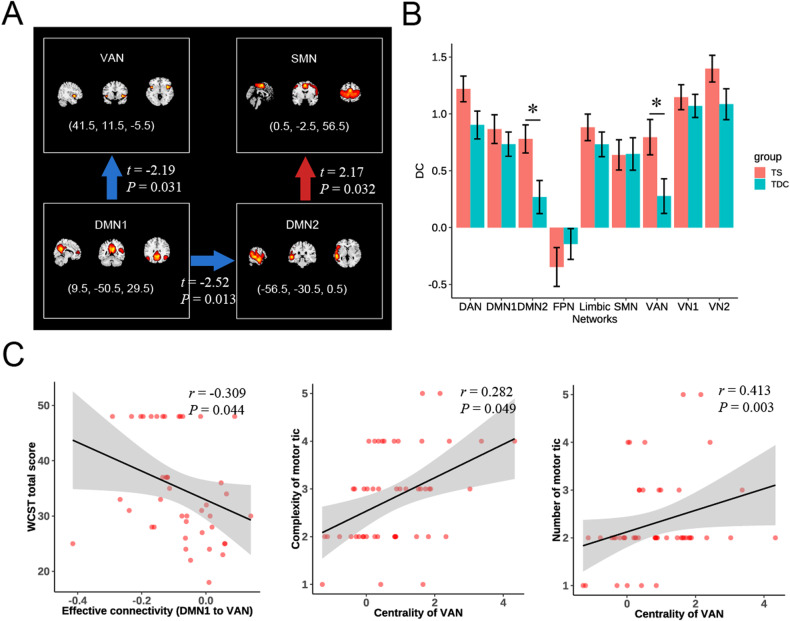
Statistical analysis for effective connectivity and degree centrality. **A** The effective connectivity (EC) showed significant group differences. The blue arrows represent stronger negative causal influence, the red arrow represent stronger positive causal influence in TS compared with TDC. **B** Group comparisons of degree centrality (DC) for each selected independent component. **C** correlations between altered EC/centrality and clinical scores. The “*” represents *P* < 0.05.

A one-sample *t* *test* of effective connectivity revealed significant positive and negative causal influences in the TS and TDC groups, respectively (Fig. S6). Compared with TDC, a positive causal influence of DMN2 on SMN increased in TS group (*t* = 2.17, *P* = 0.032), and the negative causal influence strength from DMN1 to VAN (*t* = –2.19, *P* = 0.031) and DMN2 (*t* = –2.52, *P* = 0.013) increased in the TS group (Fig. 4). Other effective connectivities didn’t show significant difference between the two groups. Correlation analysis revealed that the effective connectivity from DMN1 to the VAN was negatively correlated with the WCST total score (*r* = –0.309, *P* = 0.044). There was no significant correlation between other effective connectivities and clinical scores or duration. After accounting for head motion as a covariate, we still found significnat correlation between them (Table S4).

### Between-group differences in nodal topological metrics

We found that children with TS showed significantly increased degree centrality in DMN2 (*t* = 2.691, *P* = 0.008) and VAN (*t* = 2.363, *P* = 0.020). Children with TS also showed increased nodal efficiency in DMN2 compared with TDC (*t* = 2.085, *P* = 0.040). Other nodal topological metrics didn’t show significant group difference. Moreover, we observed that the degree centrality of the VAN was positively correlated with the complexity (*r* = 0.282, *P* = 0.049) and number of motor tics (*r* = 0.413, *P* = 0.003). No significant correlation was found between other nodal topological metrics and clinical scores or duration. The correlation results were similar to the primary results after accounting for head motion as a covariate (Table S4).

## Discussion

In this study, we found two dFC states in rfMRI scans. Children with TS showed a higher occurrence of a state characterized by strong connectivity between ICs and quicker switching between states. The FC that changed the most from state 1 to state 2 mainly included the FC between DMN and other networks. Additionally, we observed that the DMN had a stronger inhibitory influence on itself and the VAN, and that the degree centrality of the DMN and VAN increased. Moreover, some of these alterations are associated with executive function and tic symptom ratings. These findings indicated that the DMN may play an important role in abnormal state transitions, and the altered dynamic FC may be critical in the pathophysiology of TS.

### dFC state analysis

In the dFC state analysis, two recurrent functional brain states were obtained. State 1 was characterized by weak connectivity between ICs and occurred more frequently, whereas state 2 had stronger and mostly positive connectivity, and occurred at a lower frequency. Compared with healthy controls, children with TS spent more time in state 2. This finding indicates an abnormally strong connectivity during scanning. Moreover, we observed that children with TS had longer transition times than those with TDC did. Recently, Xin et al. [23] explored abnormal brain dynamic FC in 36 boys with TS compared with 27 healthy controls. Consistent with our results, they found children with TS have higher fractional time and dwell time in the state characterized by increased inter-network connections. We inferred the higher occurrence of a state with higher inter-network connectivity might be the neural substrate for the increased static connectivity in patients with TS. Consistent with this theory, previous static rfMRI studies in children with TS have found increased FC in the basal ganglia-thalamo-cortical circuits [33]. TS generally coexists with other developmental neuropsychiatric disorders such as ADHD and OCD [34]. As evidenced in the recent literature, TS shares a similar genetic background with other neurodevelopmental disorders (e.g., ADHD, OCD), eventually producing similar neuropathological alterations [35, 36]. Previous studies have found that both patients with ADHD [37] and OCD [38] show a higher number of transitions than healthy controls. Shappell et al. revealed that children with ADHD spent less time in segregated network states and more time in hyperconnected networks [39]. These results about ADHD and OCD show similarities with our results, which may explain the mechanism of comorbidity at the neuroimaging level.

To characterize the most prominent alterations from state 1 to state 2 in our study, we calculated the connectivity value difference between the two states. We found that the FC that changed most from state 1 to state 2 mainly included the FC between DMN and other networks, such as the DMN-DAN, DMN-SMN, and DMN-VN. We inferred that the alterations of FC between DMN and other networks may be important for state transitions. The DMN has been implicated in processes related to mind-wandering or task independent thoughts [40], and the integration of cognitive and emotional processes [41]. It is disrupted in a wide range of neuropsychiatric disorders including TS. Our results shows similarity with previous static FC studies about TS. Fan et al. [42] found patients with TS showed higher involvement of the dorsal medial prefrontal cortex in the connectivity of DMN and stronger coupling between DMN and left FPN compared with healthy controls. Moreover, FC within DMN correlated negatively with tic severity in patients with TS. Wen et al. [43] investigated the topological organization of the FC networks in children with TS using graph theory measures. They found children with TS showed altered topology of the DMN. Another study reported an intact DMN when adult patients with TS were instructed to lie in the scanner and were allowed to tic (compared to no tic activity), although no direct comparison with HC was made [9]. These studies together with our results highlighted role of DMN function in the pathophysiology of TS.

In children with TS, the fractional and dwell time of state 1 were positively correlated with the frequency and phonic tics, respectively. In addition, the fractional and dwell time of state 2 were negatively associated with the frequency of phonic tics. Thus, from a clinical perspective, this association may reflect a compensation mechanism in children with TS. We infer the reduced time in the loosely connected state may alleviate the phonic tics. Therefore we observed reduced time in state 1 associated with decreased symptom severity in the TS group. Recently, Ramkiran et al. [44] employed LASSO regularization using static and dynamic FC to predict tic severity in adult patients with TS. They found only the slow dynamic communication measure by the sliding-window approach significantly impacted the prediction. Their study and our correlation results indicate the importance of altered dynamic FC for tic pathophysiology.

### Effective connectivity

To further investigate which brain regions may play the most important roles in driving the whole-brain switch between different states, we conducted a mGCA. We observed that compared with healthy controls, children with TS exhibited significantly increased positive causal influence from DMN2 to the SMN, and increased negative causal influence strength from DMN1 to the VAN and DMN2. We interpreted our findings as follows: in children with TS, the DMN had a stronger inhibitory influence on itself and the VAN, as well as a stronger excitatory effect on the SMN. Similarly, Shukti et al. observed an increase in EC within the CSTC circuit [33]. Qiao et al. investigated the EC among the regions within the CSTC circuit before and after cranial electrotherapy. They found that normalization of the balance between the motor and control portions of the circuit may result in the recovery of adolescents with TS [45]. However, these published studies on EC in the TS have mainly focused on the CSTC circuit and ignored EC among other brain regions outside the circuit. Children with ADHD show widespread abnormalities in causality connectivity, particularly in attention- and memory-related regions [46]. Disrupted EC in parts of the DMN and frontostriatal network may underlie the general urge for reinforcement of compulsive behaviors [47]. These studies on ADHD and OCD may provide insights into TS.

The negative correlation between Granger causality strength from DMN1 to VAN and the WCST total score indicates that stronger inhibitory influence from DMN to VAN may lead to poorer WCST performance in children with TS. Executive functions refer to a family of top-down mental processes needed when you have to concentrate and pay attention [48]. The three core executive functions included: inhibitory control, working memory and cognitive flexibility [49]. The WCST is generally used to measure the executive function involving inhibitory control and cognitive flexibility [28]. Previous studies also showed deficit WCST performance in patients with TS [50, 51]. In summary, our findings indicate an increased causal link strength between the DMN and the VAN or SMN in children with TS, highlighting the underlying neural substrates of inhibitory control/cognitive flexibility deficits mediated by brain network interactions.

### Altered nodal topological metrics

In our study, we found that children with TS showed increased degree centrality and nodal efficiency in the DMN and increased degree centrality in VAN compared with healthy children. Similar to our results, a previous study found that patients with TS showed higher FC in the DMN than healthy controls [17]. Ramkiran et al. observed a reduction in serial information transfer (average path length) within the DMN and salience network [33]. Moreover, we observed that higher degree centrality of the VAN was associated with a higher number and complexity of motor tics. Liao et al. also found topographical alterations in the gray matter morphological networks in children with TS, including abnormal nodal degrees, betweenness, and efficiency. Some of these alterations are associated with the severity of the tic symptoms in children with TS [10]. Consistently, in our TS group, the degree centrality of the VAN was positively correlated with the number of motor tics, indicating that the higher degree centrality of the VAN in children with TS, the more serious the motor tic symptoms.

In addition, in the GCA observed in children with TS, the DMN had a stronger inhibitory influence on itself and on the VAN. Brain regions with high functional levels are likely to be more influential [30], and increased degree centrality and efficiency in the DMN in children with TS indicates a higher functional level. Therefore, we inferred that, because the DMN of children with TS had abnormally high functional levels, it exerted a stronger causal influence on other brain regions (e.g., the VAN and SMN), as we observed increased degree centrality in the VAN.

### Limitations

Our study has some limitations. First, the sample size was relatively small, perhaps providing insufficient statistical power to detect modest alterations, and the generalizability of the present results to other centers should be interpreted with caution. Second, the children with TS in our study were predominately boys. Though the higher incidence of boys [52, 53] could explain the composition of the sample in our study, further work is needed to examine sex-specific alterations of brain functional network dynamics with more samples of girls with TS. Third, we used a 1.5 T scanner in this study, which shows poorer spatial resolution and signal-to-noise ratio [54] compared with the 3 T or 7 T scanners. We have been using the 3 T MR scanner in the further study. Fourth, dFC analyses are particularly sensitive to head motion [55]. Although we applied relatively stringent inclusion criteria and found no group differences in head motion parameters, head motion remained a potential source of artifacts. Fifth, the scanning length of the rfMRI in our study was not long. A longer scan with a higher temporal resolution may provide a more refined assessment of dynamic FC features [19]. Sixth, although the sliding-window method has been widely used, the method that best captures the dynamic fluctuations remains controversial [56]. Evaluating dFC using novel methods and metrics may contribute to future advances in this field. Lastly, TS is generally comorbid with other psychiatric disorders, particularly ADHD and OCD [4]. Therefore, it would be worthwhile to investigate the potential effects of comorbidities and the TS-specific effects on functional brain alterations. In the future study, we are going to recruit isolated forms of TS, ADHD and OCD, as well as their comorbidity, to find common and disorder-specific brain dynamic functional alterations for these disorders.

## Conclusion

In summary, we found that children with TS showed a higher occurrence of a state characterized by strong connectivity between ICs and a quicker switch between the two states. The DMN may play an important role in abnormal state transitions because we observed that the FC that changed the most between the two states was between the DMN and other networks. Additionally, the DMN had increased degree centrality, efficiency and altered causal influence on other networks. Some of these alterations related to inhibitory control and tic symptom ratings may represent important aspects of pathophysiology. Our investigations of brain functional dynamics, effective connectivity, and network topological measures facilitate our understanding of the neural basis of the clinical presentation of TS.

### Supplementary information

Supplementary Materials

## Data Availability

The data that support the findings of this study are available from the corresponding authors upon reasonable request.
