# Supplementary material for: Altered dynamic functional and effective connectivity in drug-naive children with Tourette syndrome
Source: Transl Psychiatry. 2024 Jan 22;14:48. doi: 10.1038/s41398-024-02779-1 (PMC10803732; doi:10.1038/s41398-024-02779-1)
Supplement: Supplementary file 1 — Supplementary Materials [file 41398_2024_2779_MOESM1_ESM.docx]

**Supplementary Materials**

1. **Supplementary methods**

**rfMRI data preprocessing**

Preprocessing was performed using a toolbox for Data Processing & Analysis of Brain Imaging (rfmri.org/DPABI). The first 10 volumes of the rfMRI data were removed to reduce equilibration effects, leaving 140 volumes for further analysis. The remaining functional images underwent slice-timing correction and were realigned to reduce displacement between the volumes. Spatial normalization was performed using the Montreal Neurologic Institute (MNI) brain template, each voxel was resampled to 3 × 3 × 3 mm^3^, and spatial smoothing was performed using a 6 mm full-width half-maximum Gaussian kernel. To eliminate the influence of head motion, participants with a maximum displacement of > 3 mm and maximum rotation of > 3° were excluded from further analysis.

**GICA and identification of independent components (ICs)**

Group independent component analysis (GICA) is a data-driven approach for decomposing rfMRI data into functionally homogeneous regions [1]. GICA enables whole-brain analysis without resorting to atlas-defined regions of interest that may merge or delineate functionally distinct areas [2]. First, we performed a principal component analysis for subject-specific data, in which the principal components were identified using standard economy-size decomposition. To identify the intrinsic connectivity networks, we performed spatial GICA using the GIFT toolbox (Mialab.mrn.org/software/gift). In data reduction for group comparisons, the concatenated subject-reduced data were decomposed into an estimated 21 ICs. The reliability and stability of the Informax GICA algorithm in ICASSO [3] were ensured by repeating the algorithm 20 times, and using the most central run to reconstruct subject-specific time courses and spatial maps for each IC using the GICA back reconstruction algorithm [4]. ICs with a quality index (*I*_q_) ≥ 0.8 were selected for further analysis, while ICs with *I*_q_ < 0.8 were discarded [5]. Using a one-sample *t*-*test* across all subjects for each IC, we obtained a spatial map (*t*-map) for each IC with a threshold of *t* > mean (*μ*) + 4SD (*σ*) [6].

The selected ICs should also meet the following criteria [7]: the peak coordinates of spatial maps located in the gray matter, with minimal spatial overlap with white matter, vessels, cerebrospinal fluid, ventricles, or susceptibility artifacts, and time courses characterized by a high dynamic range. Furthermore, we also visually confirmed IC linkages to brain networks by establishing that the peak coordinates of ICs were located within the template for this network. ICs that did not meet these criteria were excluded from our study. Finally, nine ICs were identified as meaningful.

Based on Yeo’s seven functional brain network templates [8], we sorted the nine selected ICs into different functional networks (Figure 1): dorsal attention network (DAN), DMN, FPN, Limbic network, SMN, ventral attention network (VAN), and visual network (VN). In particular, we used the “Sorting Components” module within the GIFT toolbox to classify the ICs onto Yeo’s functional brain network templates. The spatial sorting classifies the components by comparing the component’s image with the templates by using the Multiple Linear Regression (MLR) method. We used the coefficient of determination (*R*^2^) between the spatial maps of ICs in our study and Yeo’s functional brain network templates [8] as the criterion for selecting ICs. A higher *R*^2^ indicates a higher similarity between an IC and a specific network of the template. Each IC was assigned to the network with the highest *R*^2^. We also used the Stanford functional ROI template [9] and the Anatomical Automatic Labeling (AAL) templates to replicate and validate the analysis.

**dFC state analysis**

dFC was examined using the temporal dFNC toolbox in the GIFT. Before computation for windowed matrices, additional post-processing steps were performed for the time courses of all ICs to regress out the influence of noise sources, including: 1) detrending (regressing linear, quadratic, and cubic trends); 2) despiking using 3D DESPIKE; 3) low-pass filtering using a high-frequency cut-off of 0.15 Hz; and 4) reducing the influence of six parameters of head movement using regression.

A sliding-window approach was used to explore time-varying changes in FC within the nine ICs identified during the rfMRI scans. We chose a 20-TR window (60 s) because previous studies have suggested that windows of 30–60 s can successfully capture the patterns of resting-state fluctuations in the dFC [10]. We used a Gaussian (*σ* = 3 TRs) function to create a tapered window, slide stepwise by one TR along the scan image time series, and then computed the 9 × 9 pairwise FC matrix using Pearson’s correlations in every sliding window. The correlation values of the pairwise functional matrices were converted to *z*-values using Fisher’s *z*-transformation to improve the normality and comparability.

To assess recurring dFC patterns, k-means clustering was performed on the FC matrices of all the time windows for all individuals. The k value was varied from two to 10 to determine the optimal value. Using the silhouette coefficient, the optimal k value was determined to be two (Figure S1). We used the cluster centroids of all the participants to represent the two recurring FC states (Figure 2C). To visualize the dFC states in the two groups, we estimated group-specific cluster centroids (Figure 2A and B). To examine the temporal properties of the dFC states, we assessed three different state characteristics: (1) the fractional time, which indicates the proportion of the time window belonging to each state; (2) the mean dwell time, which represents the average length of time spent in each state (measured number of consecutive time windows) before switching to other states; and (3) the number of transitions, which represents the number of switches among states over time. To ensure consistency and validity of the dFC state analysis at different window sizes, we used 15-TR to 19-TR window size to repeat the above analysis and validate the results.

**2. Supplementary results**

**2.1 Validation analysis**

**2.1.1 Using different window size**

Using 15-TR to 19-TR window size, two recurring brain functional states were defined. The centroids under 15-TR window size were shown in Figure S3. Across all participants, state 1 was characterized by weak connectivity between ICs, and the occurrence proportion of state 1 was 70%; state 2 was characterized by stronger and mostly positive connectivity in general, and the occurrence proportion of state 2 was 30%.

Group comparisons of state transition metrics showed similar results with the main results. Under -15TR window size children with TS showed decreased fractional time in state 1 (*P* < 0.001, *P*_FDR_ < 0.001), and increased fractional time in state 2 (*P*_FDR_ = 0.000) compared with TDC. We also found that children with TS exhibited lower dwell time in state 1 (*P* < 0.001, *P*_FDR_ < 0.001), and higher dwell time in state 2 (*P* = 0.002, *P*_FDR_ = 0.003) than TDC. Moreover, we observed higher number of transitions of children with TS than TDC (*P* = 0.015, *P*_FDR_ = 0.015). In the correlation analysis, we found that the frequency of phonic tics was positively correlated with the fractional time of state 1 (*r* = 0.374, *P* = 0.042) and negatively correlated with the fractional time of state 2 (*r* = – 0.374, *P* = 0.042) in the TS group. The results under 15-TR window size were shown in Figure S3. The group comparison results of 16-TR to 19-TR were shown in Table S3.

**2.1.2 Using different templates**

The Stanford functional ROI template ([findlab.stanford.edu/functional_ROIs.html](http://findlab.stanford.edu/functional_ROIs.html)) and the Anatomical Automatic Labeling (AAL) templates were used to replicate and validate the dynamic functional connectivity results. Using the Stanford functional ROI template, eight ICs were identified as meaningful based on the criteria described in the main text. We sorted the eight selected ICs into the following functional networks for further analysis: default mode network (DMN), executive control network (ECN), language network (LAN), salience network (SAN), sensorimotor network (SMN), and visual network (VN). We have also used the AAL templates to segment brain regions directly to replicate the primary analysis. The validation analysis showed similar results to the main results (Figures S5 and S6). We obtained almost the same results as the primary ones using the Stanford templates. When using the AAL templates, we obtained similar results. The group comparison results using the AAL templates were significant but didn’t survive the FDR correction, and though some of the results were not significant but had the same trend of alterations as the primary results.

**2. Supplementary Tables**

**Table S1.** There was no significant correlation between clinical characteristics and head motion in children with Tourette Syndrome.

| Clinical Scores | *r* value | *P* value |
| --- | --- | --- |
| Duration (months) | 0.057 | 0.738 |
| Total YGTSS score | -0.149 | 0.288 |
| Total motor tic score | -0.245 | 0.074 |
| Number motor tic | -0.195 | 0.180 |
| Frequency of motor tic | -0.070 | 0.632 |
| Strength of motor tic | -0.016 | 0.913 |
| Complexity of motor tic | -0.286 | 0.091 |
| Interference of motor tic | -0.101 | 0.492 |
| Total phonic tic score | 0.031 | 0.824 |
| Number phonic tic | -0.243 | 0.196 |
| Frequency of phonic tic | 0.092 | 0.630 |
| Strength of phonic tic | 0.039 | 0.839 |
| Complexity of phonic tic | -0.046 | 0.811 |
| Interference of phonic tic | 0.122 | 0.521 |
| WCST total number of correct | 0.048 | 0.759 |

**Abbreviations:** YGTSS, Yale Global Tic Severity Scale; WCST, Wisconsin Card Sorting Test.

**Table S2.** Mathematical definitions and interpretations of nodal topological network measures used in the present study.

| Measures | Definitions |
| --- | --- |
| Nodal degree centrality (*k*) | Degree centrality is a simple measurement of the connectivity of a node with the rest of nodes in a network. In a network *G* with *N* nodes and *K* edges, the degree *k_i_* of node *i* is defined as:  *k_i_=*$\sum_{jG} a_{ij}$  where *a_ij_* is the *i* th row and *j* th column element of the adjacency matrix *A*. The degree has a straightforward neurobiological interpretation: nodes with a high degree are interacting, structurally or functionally, with many other nodes in the network [11]. |
| Nodal betweenness centrality (*B*) | The nodal betweenness centrality for a given node characterizes its effect on information flow between other nodes. Nodal betweenness centrality is the fraction of all shortest paths in the network that contain a given node. The nodal betweenness centrality of node *i* is computed as [12]:  *B_i_=*$\sum_{s\neq it} \frac{\sigma_{st}(i)}{\sigma_{st}}$  where $\sigma_{st}$ is the total number of shortest paths from node *s* to node *t*, and $\sigma_{st}(i)$ is the number of those paths that pass through node *i*. |
| Nodal clustering coefficient (*C*) | The cluster coefficient of a given node measures the likelihood its neighborhoods are connected to each other. For a graph *G* = (*V, E*), the clustering coefficient of node *i* is computed as [13]:  *C_i_=*$\frac{\vert\left\{ e_{jk}: v_{j}, v_{k}\in N_{i,}e_{jk} \in E \right\}\vert}{k_{i}(k_{i}-1)}$  where $N_{i}$is defined as its immediately connected neighbors. |
| Nodal efficiency (*e*) | Nodal efficiency measures the information propagation ability of a node with the rest of the nodes in the network. The nodal efficiency of node *i* is computed as:  *e_i_* _=_ $\frac{1}{(N-1)}\sum_{j\neq iG} \frac{1}{d_{ij}}$  where *d_ij_* is the shortest path length between node *i* and node *j*, and *N* is the number of nodes in the network [14]. |
| Nodal local efficiency ($e_{loc})$ | The local efficiency for a given node measures how efficient the communication is among the first neighbors of this node when it is removed. Which can be computed as follows [15]:  $e_{loc}(i)=\sum_{i \in G} Eglobal(G_{i})$  where $Eglobal(G_{i})$ is the global efficiency of the sub-graph $G_{i}$. |

**Table S3-1.** The validation analysis using 15-TR window size.

|  | TS  Median (Quartile) | TDC  Median (Quartile) | *P* value | *P_FDR_* value |
| --- | --- | --- | --- | --- |
| FT1 | 0.712(0.380) | 0.860(0.310) | < 0.001 | < 0.001 |
| FT2 | 0.288(0.380) | 0.140(0.310) | < 0.001 | < 0.001 |
| DT1 | 25.167(26.250) | 39.333(63.792) | < 0.001 | < 0.001 |
| DT2 | 16.000(14.542) | 8.350(12.400) | < 0.001 | < 0.001 |
| NT | 4.000(3.000) | 3.500(4.000) | 0.012 | < 0.001 |

**Table S3-2.** The validation analysis using 16-TR window size.

|  | TS  Median (Quartile) | TDC  Median (Quartile) | *P* value | *P_FDR_* value |
| --- | --- | --- | --- | --- |
| FT1 | 0.690(0.391) | 0.887(0.286) | < 0.001 | < 0.001 |
| FT2 | 0.310(0.391) | 0.113(0.286) | < 0.001 | < 0.001 |
| DT1 | 26.667(26.175) | 36.500(36.438) | 0.007 | 0.009 |
| DT2 | 13.750(19.000) | 7.250(9.625) | < 0.001 | < 0.001 |
| NT | 4.000(3.000) | 4.000(4.000) | 0.153 | 0.153 |

**Table S3-3.** The validation analysis using 17-TR window size.

|  | TS  Median (Quartile) | TDC  Median (Quartile) | *P* value | *P_FDR_* value |
| --- | --- | --- | --- | --- |
| FT1 | 0.272(0.394) | 0.098(0.299) | < 0.001 | < 0.001 |
| FT2 | 0.728(0.394) | 0.902(0.299) | < 0.001 | < 0.001 |
| DT1 | 16.250(14.417) | 9.000(14.125) | < 0.001 | < 0.001 |
| DT2 | 28.500(30.813) | 55.000(94.250) | < 0.001 | < 0.001 |
| NT | 4.000(3.000) | 2.000(4.000) | 0.011 | 0.011 |

**Table S3-4.** The validation analysis using 18-TR window size.

|  | TS  Median (Quartile) | TDC  Median (Quartile) | *P* value | *P_FDR_* value |
| --- | --- | --- | --- | --- |
| FT1 | 0.725(0.391) | 0.898(0.307) | < 0.001 | < 0.001 |
| FT2 | 0.275(0.391) | 0.102(0.307) | < 0.001 | < 0.001 |
| DT1 | 27.708(30.688) | 64.250(93.833) | < 0.001 | < 0.001 |
| DT2 | 16.250(14.167) | 9.000(14.125) | < 0.001 | < 0.001 |
| NT | 4.00(4.00) | 2.00(4.00) | 0.012 | 0.012 |

**Table S3-5.** The validation analysis using 19-TR window size.

|  | TS  Median (Quartile) | TDC  Median (Quartile) | *P* value | *P_FDR_* value |
| --- | --- | --- | --- | --- |
| FT1 | 0.744(0.450) | 0.917(0.281) | < 0.001 | < 0.001 |
| FT2 | 0.256(0.450) | 0.083(0.281) | < 0.001 | < 0.001 |
| DT1 | 30.667(32.250) | 53.500(88.333) | < 0.001 | < 0.001 |
| DT2 | 16.833(14.333) | 7.500(15.000) | < 0.001 | < 0.001 |
| NT | 3.000(3.000) | 2.000(4.000) | 0.029 | 0.029 |

**Abbreviations:** FT1, fractional time in state 1; FT2, fractional time in state 2; DT1, dwell time in state 1; DT2, dwell time in state 2; NT, number of transitions.

**Table S4.** After accounting for head motion as a covariate, we found similar correlation results to the primary results.

| Correlation | *r* value | *P* value |
| --- | --- | --- |
| FT1 - Frequency of the phonic tics | 0.384 | 0.045* |
| FT2 - Frequency of the phonic tics | -0.384 | 0.045* |
| DT1 - Strength of the phonic tics | 0.351 | 0.060 |
| DT2 - Strength of the phonic tics | -0.351 | 0.060 |
| EC of DMN1 to VAN – WCST total score | -0.307 | 0.048* |
| Centrality of VAN – Number of motor tic | 0.269 | 0.051 |
| Centrality of VAN – complexity of motor tic | 0.330 | 0.018* |

**Abbreviations:** FT1, fractional time in state 1; FT2, fractional time in state 2; DT1, dwell time in state 1; DT2, dwell time in state 2; EC, effective connectivity; WCST, Wisconsin Card Sorting Test; VAN, ventral attention network.

**3. Supplementary Figures**

**Figure S1.** K-means clustering analysis assessing the reoccurring dynamic functional connectivity patterns. According to the maximum silhouette coefficient, the optimal cluster number was determined to be two.

**
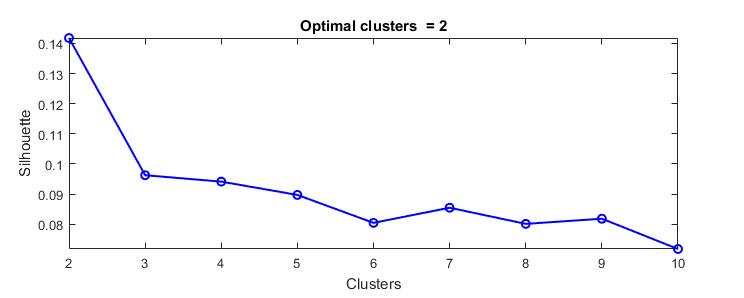
**

**Figure S2.** Group specific cluster centroids and cluster centroids for all participants under 15-TR window size are similar with that under 20-TR window size. (A) Cluster centroids for Tourette’s syndrome (TS) group. (B) Cluster centroids for typically developing children (TDC) group. (C) Cluster centroids for all participants.


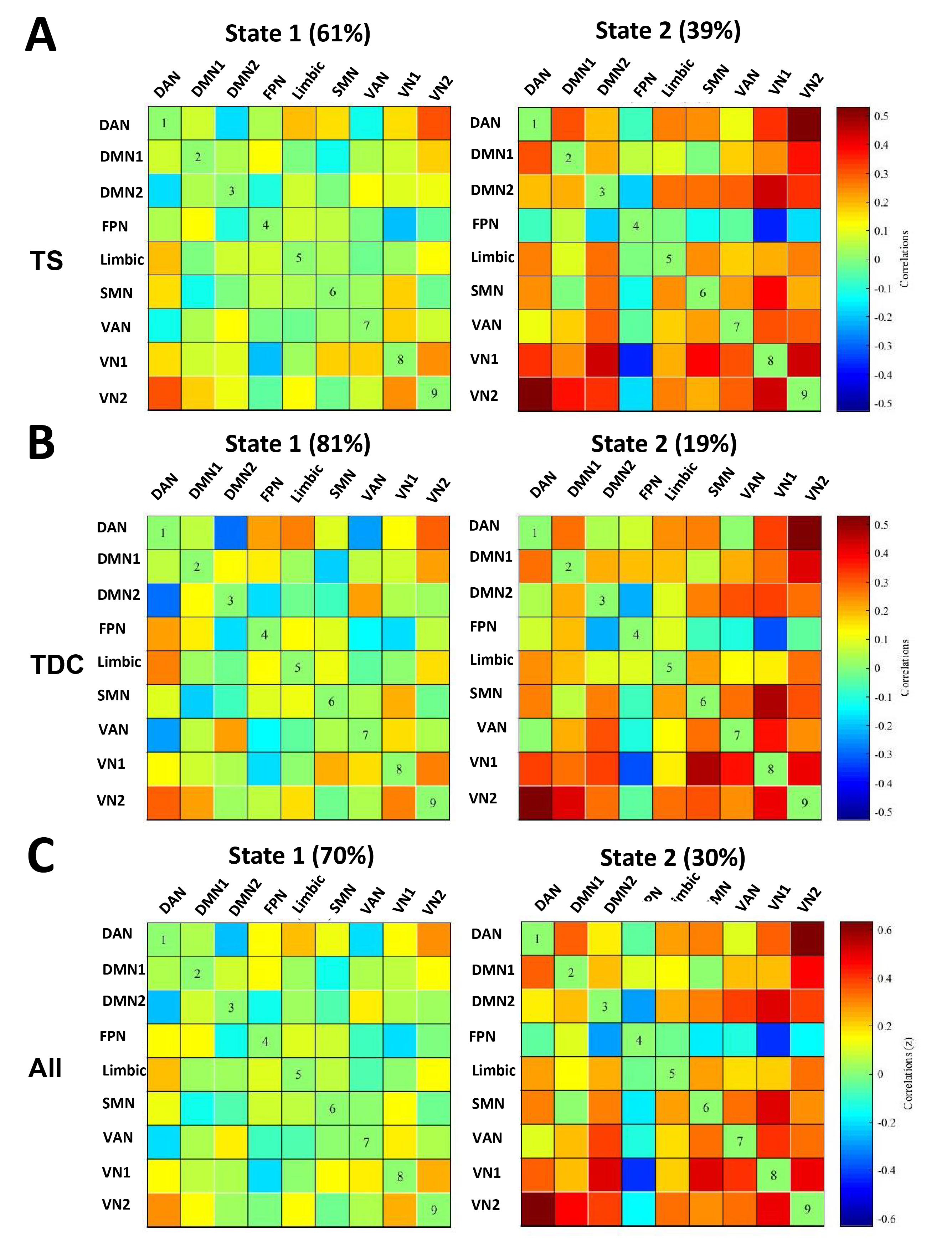


**Figure S3.** The validation analysis using 15-TR window size showed similar results with the main results. (A) Group comparisons of state transition metrics under 15-TR window size. (B) The correlations between state transition metrics and tic symptom ratings. The “**” represents *P*_FDR_ < 0.05.


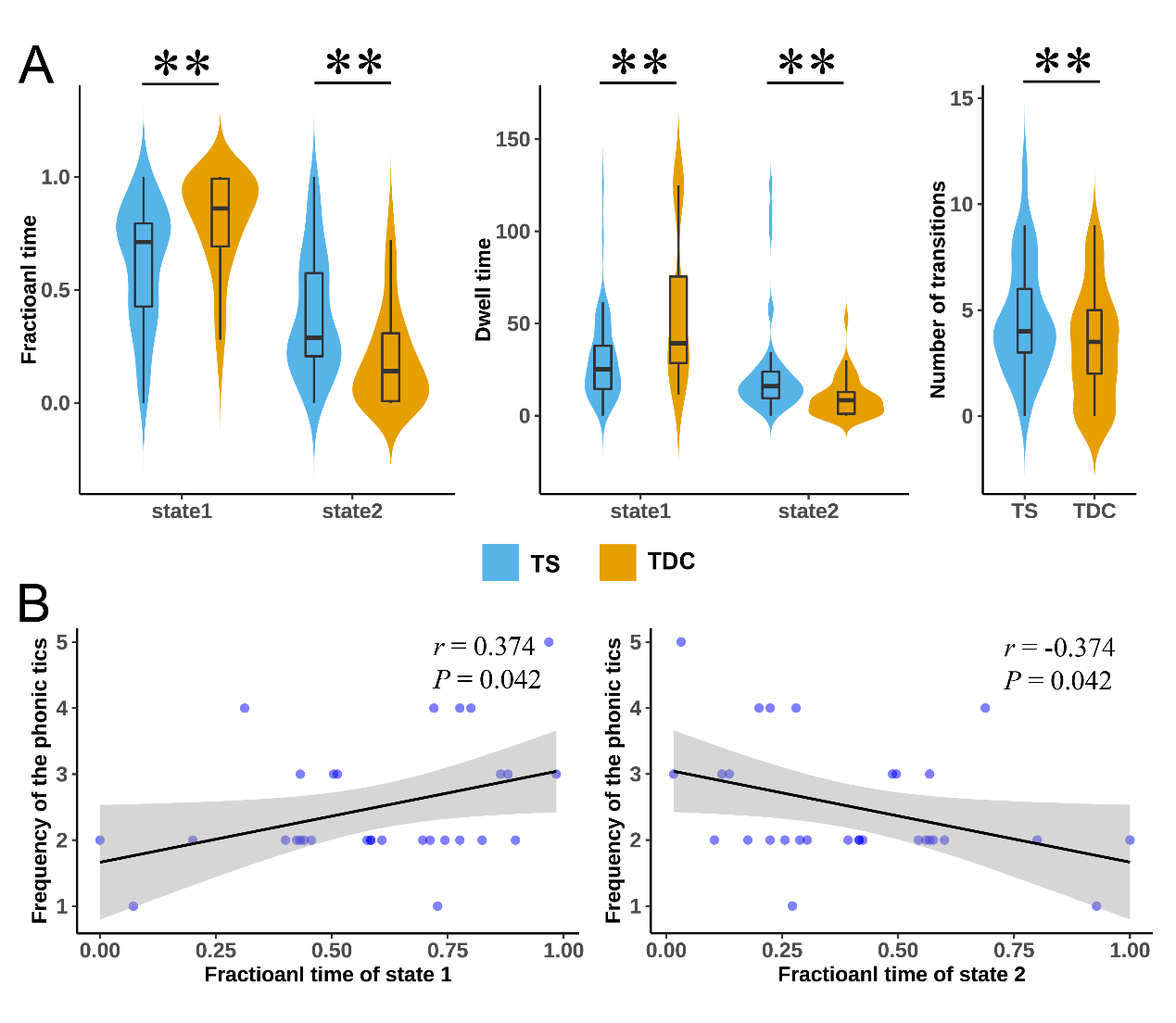


**Figure S4.** The validation analysis using the Stanford templates with 20-TR window size showed similar results to the main results. (A) The two states when using the Stanford template, the cluster centroids for the two states were similar with that using the Yeo’s template. (B) Group comparisons of state transition metrics. The “**” indicates *P*_FDR_ < 0.05.


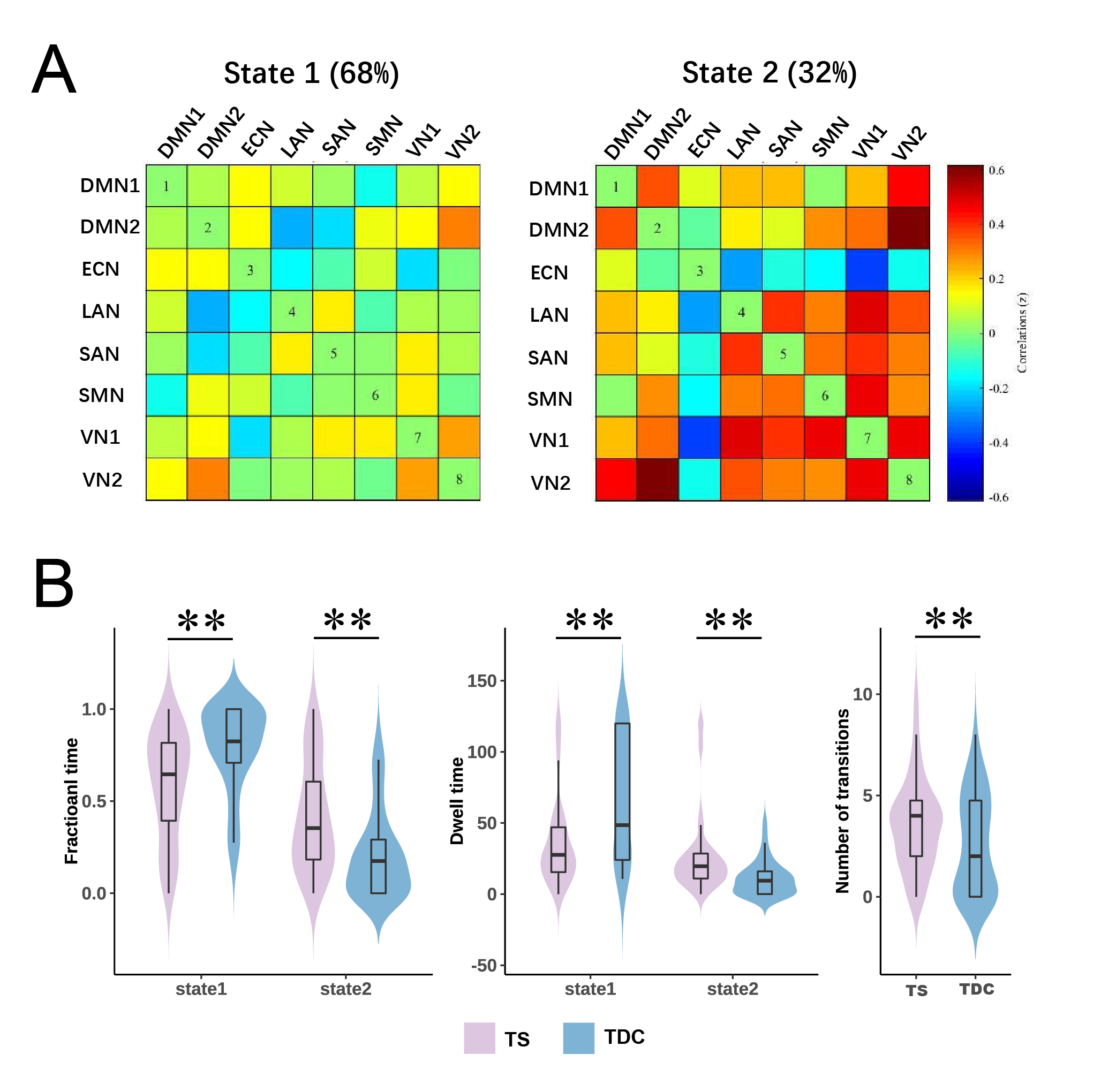


**Figure S5.** The validation analysis using the Anatomical Automatic Labeling (AAL) templates showed similar results to the main results. (A) The two states when using the Stanford template, the cluster centroids for the two states were similar with that using the Yeo’s template. (B) Group comparisons of state transition metrics. The “*” indicates *P* < 0.05.


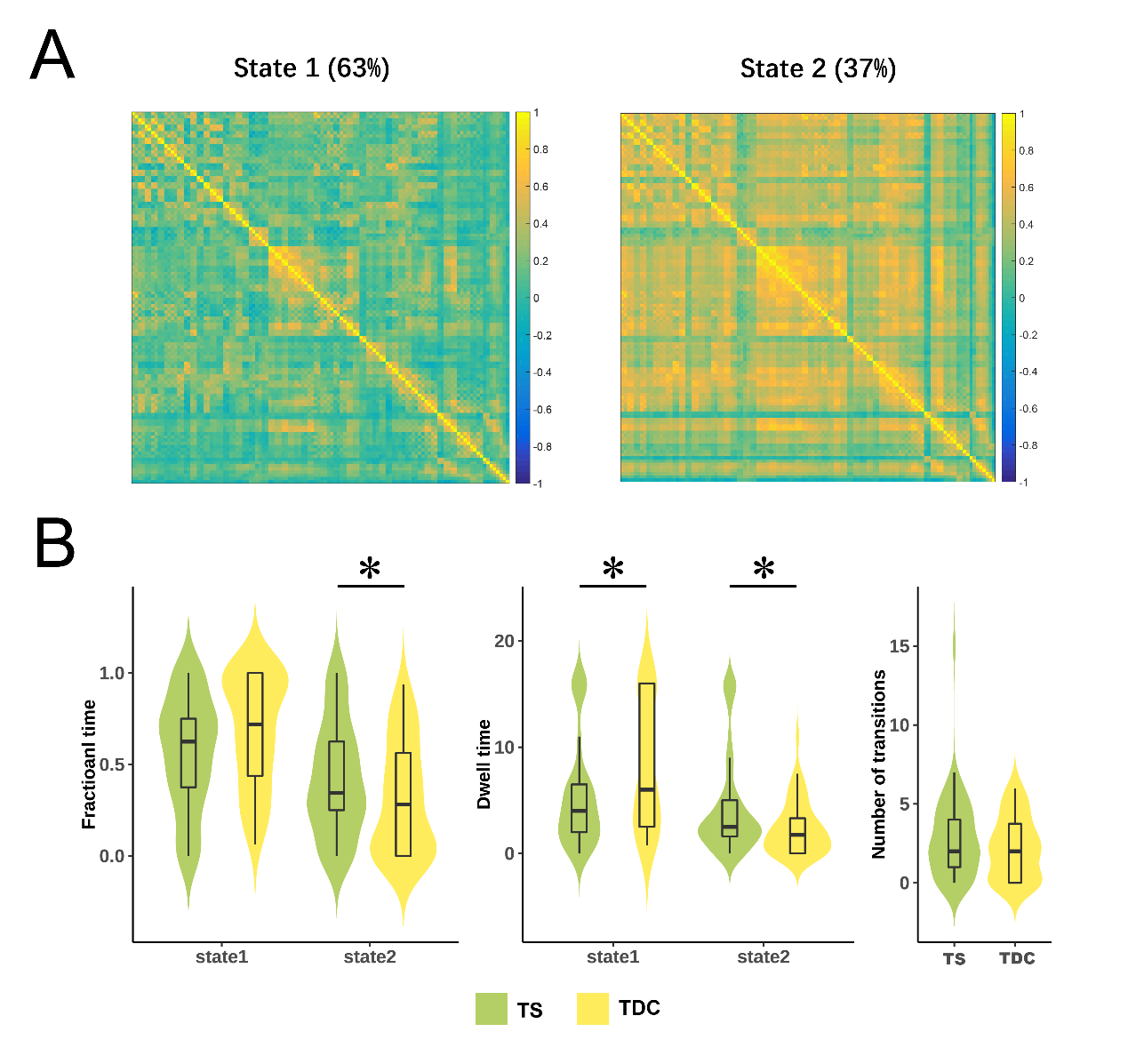


**Figure S6.** Effective connectivity among the nine ICs in the groups of TS (A, B, C) and TDC (D, E, F). (A, D) The significant positive causal influence in TS and TDC groups, respectively (one-sample *t*-test, *P* < 0.05). (B, E) The significant negative causal influence in TS and TDC groups, respectively (one-sample *t*-test, *P* < 0.05). (C, F) The group mean Granger causality strength map in TS and TDC groups, respectively. Cells colored in warm colors represent positive causal influence, across nodes (column to row), while cells colored in cold colors represent negative causal influence.


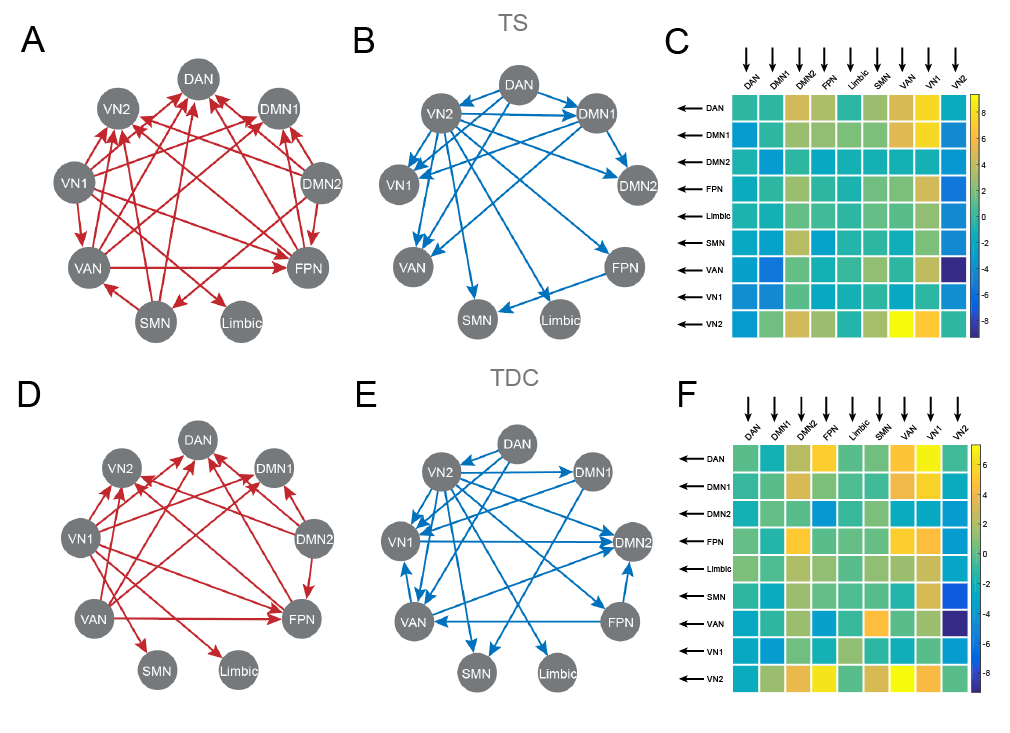


**References**

1. Kiviniemi V, Starck T, Remes J, Long XY, Nikkinen J, Haapea M *et al.* Functional Segmentation of the Brain Cortex Using High Model Order Group PICA. *Human Brain Mapping* 2009; **30**(12)**:** 3865-3886.

2. Allen EA, Erhardt EB, Wei Y, Eichele T, Calhoun VD. Capturing inter-subject variability with group independent component analysis of fMRI data: a simulation study. *Neuroimage* 2012; **59**(4)**:** 4141-4159.

3. Himberg J, Hyvarinen A, Esposito F. Validating the independent components of neuroimaging time series via clustering and visualization. *Neuroimage* 2004; **22**(3)**:** 1214-1222.

4. Calhoun VD, Adali T, Pearlson GD, Pekar JJ. A method for making group inferences from functional MRI data using independent component analysis. *Hum Brain Mapp* 2001; **14**(3)**:** 140-151.

5. Ma S, Correa NM, Li XL, Eichele T, Calhoun VD, Adali T. Automatic Identification of Functional Clusters in fMRI Data Using Spatial Dependence. *Ieee T Bio-Med Eng* 2011; **58**(12)**:** 3406-3417.

6. Allen EA, Erhardt EB, Damaraju E, Gruner W, Segall JM, Silva RF *et al.* A baseline for the multivariate comparison of resting-state networks. *Front Syst Neurosci* 2011; **5:** 2.

7. Allen EA, Damaraju E, Plis SM, Erhardt EB, Eichele T, Calhoun VD. Tracking whole-brain connectivity dynamics in the resting state. *Cereb Cortex* 2014; **24**(3)**:** 663-676.

8. Yeo BT, Krienen FM, Sepulcre J, Sabuncu MR, Lashkari D, Hollinshead M *et al.* The organization of the human cerebral cortex estimated by intrinsic functional connectivity. *J Neurophysiol* 2011; **106**(3)**:** 1125-1165.

9. Shirer WR, Ryali S, Rykhlevskaia E, Menon V, Greicius MD. Decoding subject-driven cognitive states with whole-brain connectivity patterns. *Cereb Cortex* 2012; **22**(1)**:** 158-165.

10. Preti MG, Bolton TA, Van De Ville D. The dynamic functional connectome: State-of-the-art and perspectives. *Neuroimage* 2017; **160:** 41-54.

11. Rubinov M, Sporns O. Complex network measures of brain connectivity: uses and interpretations. *Neuroimage* 2010; **52**(3)**:** 1059-1069.

12. Brandes U. A faster algorithm for betweenness centrality. *Journal of Mathematical Sociology* 2001; **25**(2)**:** 163-177.

13. Fagiolo G. Clustering in complex directed networks. *Physical Review E* 2007; **76**(2).

14. Achard S, Bullmore E. Efficiency and cost of economical brain functional networks. *PLoS Comput Biol* 2007; **3**(2)**:** e17.

15. Chen HF, Huang LL, Yang D, Ye Q, Guo MD, Qin RM *et al.* Nodal Global Efficiency in Front-Parietal Lobe Mediated Periventricular White Matter Hyperintensity (PWMH)-Related Cognitive Impairment. *Frontiers in Aging Neuroscience* 2019; **11**.
